# Probabilistic visual and electromagnetic data fusion for robust drift-free sequential mosaicking: application to fetoscopy

**DOI:** 10.1117/1.JMI.5.2.021217

**Published:** 2018-02-22

**Authors:** Marcel Tella-Amo, Loic Peter, Dzhoshkun I. Shakir, Jan Deprest, Danail Stoyanov, Juan Eugenio Iglesias, Tom Vercauteren, Sebastien Ourselin

**Affiliations:** a University College London, Wellcome/EPSRC Center for Interventional and Surgical Sciences, London, United Kingdom; b KU Leuven, Center for Surgical Technologies, Faculty of Medicine, Leuven, Belgium; c University College London, Translational Imaging Group, CMIC, Medical Physics, London, United Kingdom

**Keywords:** fetoscopy, laser coagulation, twin-to-twin transfusion syndrome, sequential mosaicking, globally consistent mosaicking, electromagnetic tracking, sensor fusion

## Abstract

The most effective treatment for twin-to-twin transfusion syndrome is laser photocoagulation of the shared vascular anastomoses in the placenta. Vascular connections are extremely challenging to locate due to their caliber and the reduced field-of-view of the fetoscope. Therefore, mosaicking techniques are beneficial to expand the scene, facilitate navigation, and allow vessel photocoagulation decision-making. Local vision-based mosaicking algorithms inherently drift over time due to the use of pairwise transformations. We propose the use of an electromagnetic tracker (EMT) sensor mounted at the tip of the fetoscope to obtain camera pose measurements, which we incorporate into a probabilistic framework with frame-to-frame visual information to achieve globally consistent sequential mosaics. We parametrize the problem in terms of plane and camera poses constrained by EMT measurements to enforce global consistency while leveraging pairwise image relationships in a sequential fashion through the use of local bundle adjustment. We show that our approach is drift-free and performs similarly to state-of-the-art global alignment techniques like bundle adjustment albeit with much less computational burden. Additionally, we propose a version of bundle adjustment that uses EMT information. We demonstrate the robustness to EMT noise and loss of visual information and evaluate mosaics for synthetic, phantom-based and *ex vivo* datasets.

## 1 Introduction

Twin-to-twin transfusion syndrome (TTTS) complicates 10% to 15% of monochorionic diamniotic pregnancies.^1^ Monochorionic twins share a single placenta and their circulation due to the presence of intertwin anastomes. A certain unfavorable pattern may result in an imbalance of intertwin blood flow, leading to acute, mid-trimester TTTS. It causes overproduction of urine (hence polyhydramnios) in the recipient, whereas the other fetus will have oligohydramnios. Due to the acute overdistention of the uterus, mothers may go into labor or rupture their membranes. TTTS can also lead to cardiac dysfunction in one or both fetuses, worsening the prognosis. If this condition is not treated, then the outcome is nearly always fatal.^2^

The standard of care today is fetoscopic laser photocoagulation,^3^ which has been shown to be more effective than serial removal of excessive amniotic fluid.^4^ The procedure consists of the insertion of a fetoscope, identification and coagulation of all visible anastomoses, and functional disconnect the two circulations.

The success of this operation is dependent on many variables, some of them related to the operative technique. The surgeon has to be able to inspect as much of the placenta and understand its angioarchitecture. Ideally, one obtains a general insight on the nature (whether the connecting vessels are arteries or veins) and location of all anastomoses. Following this, first arteriovenous and then venoarterial connections are coagulated. At the end of the procedure, it is recommended to superficially laser the area between the lasered anastomoses to avoid the persistence of flow over nonvisualized, smaller anastomoses (referred to as the Solomon or bichorionization technique^2^). Fetoscopy is typically performed with 1.3 to 2.0 mm fiberendoscope and limited light. The most limiting factor for keeping an overview of the vascular anatomy is the small field-of-view.

To address this limitation, the creation of a 2-D mosaic of the placenta has been proposed^5^^–^^7^ as a means of expanding the field-of-view by stitching the fetoscopic images to a common reference frame.

The number of images needed to cover the whole placenta is an additional challenge. Clinical imaging conditions are also restrictive due to a lack of visual texture and color contrast between arteries and veins, in particular of small diameter, and visual artifacts, such as blood, amniotic fluid particles, the presence of the intertwin membrane, or even fetal movements, which may perturb the vision. To leverage the use of imagery, there should be a large overlap between adjacent images to ensure a successful registration at the expense of increasing, even more, the number of frames.

**Fig. 1:**
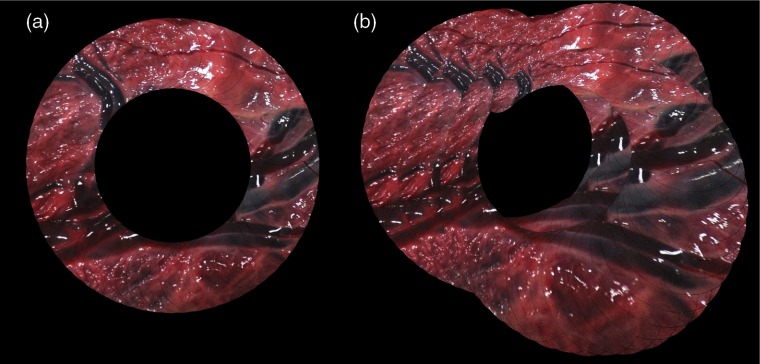
Mosaics of 200 synthetic images where the camera has moved following a circular pattern of four laps. (a) The ground truth mosaic. (b) A mosaic with drift due to the accumulation of error in subsequent iterations.

Local vision-based mosaicking algorithms make use of pairwise transformations between images to compose a mosaic. This has a fundamental limitation; since the transformation of each frame to the reference space is made dependent of all the previous pairwise registrations, any new pairwise registration error is propagated through a chain of transformations. As a result, an inevitable, progressive drift in the reference space occurs, which can even degenerate in a rupture of the chain of transformation if a pair of images cannot be registered. In order to illustrate this effect on a simulated dataset, Fig. 1(a) shows a ground truth mosaic composed of 200 images, where the camera has moved following a circular pattern. Figure 1(b) shows a mosaic that has experienced drift due to the composition of homographies.

To address this problem, we propose the use of an electromagnetic tracker (EMT) system by attaching an EMT sensor^8^^–^^13^ to the tip of the fetoscope. The EMT system provides measurements of the 3-D pose of the sensor, which then relate to the 3-D pose of the fetoscope through a precomputed rigid hand–eye calibration matrix. These measurements do not provide enough information to create a mosaic since the geometry of the scene is unknown. In fact, even if the geometry of the scene was known, the noise or jitter in these measurements would propagate to the mosaic space, resulting in misregistrations in the mosaic. Hence, the generation of the mosaic using exclusively the EMT information is infeasible (see Sec. 4.2.2). However, the fusion of these measurements is extremely valuable in order to guide the estimation of the mosaic and prevent it from drifting; especially in fetoscopy, where the poor quality of the pairwise registrations can accentuate the drift.

In this paper, we present a probabilistic model that uses the complementarity between the EMT and visual information to drive the estimation toward globally consistent mosaics, i.e., that do not suffer from drift, independently of the number of frames. Additionally, we compare our algorithm with the state-of-the-art in global alignment,^14^^,^^15^ i.e., bundle adjustment, and show that we achieve a similar performance to the state-of-the-art with a much lower computational burden.

The improvement in terms of computational complexity is mostly observed in two main steps: the matching and the nonlinear optimization. Since in bundle adjustment, the number of image pairs where matching needs to be attempted is $N^{2}$ in the worst case, where N is the number of images in the sequence, this stage of the algorithm has a complexity of $O(N^{2})$. In the proposed algorithm, only the images within a fixed window are considered for matching. Therefore, only a fixed number of images proportional to N is matched (O(N)). Analogously, the visual cost function computes the residuals for all the obtained correspondences in potentially $N^{2}$ pairs of images [$O(N^{2})$] and estimates all the parameters at a time while our algorithm computes only the visual residual within the window [O(N)] optimizing only for a small subset of parameters.

Finally, we show that our algorithm is robust to EMT noise and loss of visual information and evaluate mosaics for synthetic and phantom-based datasets. Additionally, we propose a version of bundle adjustment that incorporates EMT information in an analogous manner.

The rest of the paper is structured as follows. In Sec. 2, we discuss the state-of-the-art in drift-free mosaicking using visual information as well as sensor fusion. Section 3 details our approach and Sec. 4 provides an experimental evaluation. Finally, in Sec. 5, we discuss the results and the limitations of the approach, and we propose future research lines to overcome them.

## 2 Related Work

Mosaicking algorithms have been extensively explored in computer vision for the last two decades. Vision-based mosaicking generally relies on estimating transformations with respect to the mosaic space by chaining transformations between adjacent images.^14^^–^^16^ Therefore, it inherently accumulates drift. Additionally, if one of the transformations fails to be estimated, the resulting mosaic cannot be computed further.

Michaelsen^17^ observed that a translation-based mosaic accumulates less drift since no multiplicative effect applies. He presented a patchwise algorithm, where the normal vector to the imaged patch is used to correct the homography between the first and last image in the patch such that only x-y translation components are used to stitch the patch into the global reference frame.

As an alternative strategy, Sawhney et al.^18^ explored the idea of iteratively finding the topology of the images. First, using a translation-based registration, the authors check for overlapping images to determine the neighborhood of a given image. Then, they use a projective mapping to estimate locally consistent patches within the neighborhood. Finally, a global refinement of the mosaic is performed to avoid local minima due to the local refinement of the patches. Given that the EMT system provides the position of the cameras, we directly have information about the topology and we aim for a sequential estimate that is globally consistent.

Given that the accumulation of error leads to nonmatching image positions when revisiting the same scene, so-called loop-closing strategies propose to identify and add the loop closure overlapping frames as an additional constraint^19^^–^^21^ and then correct for the accumulation of error in the rest of the loop. Civera et al.^22^ keep track of the observed features and optimize for all the camera poses in every iteration, having a natural loop closing effect since the whole loop is optimized for. This has the additional cost of having to compare all images to a growing map in each iteration. The creation of this map is not trivial in our scenario given the poor fetoscopic image quality: the lack of visual texture and color contrast between arteries and veins, amniotic fluid particles, and the presence of the intertwin membrane and fetal movements.

Another cause of this accumulation of error in a planar scenario is over-parametrization.^23^ In a planar scenario, the family of homographies that defines the motion of a monocular camera can be minimally parametrized by six parameters for each camera pose and the three global parameters representing the plane, whereas in classic mosaicking, every pairwise relation is parametrized by a full homography. There exists a restricted group of homographies that can each be decomposed into the camera motion and global plane. This decomposition has been used by several authors, for example, Malis and Benhimane^24^ used it for visual servoing and some authors used it for mosaicking as well.^7^^,^^17^^,^^23^^,^^25^

Closely related to the last idea, Olsson and Eriksson^26^ showed an increased performance in the estimation of the plane by minimizing the reprojection errors as a function of the plane as opposed to using a plane fitting procedure after triangulating the 3-D points. Given that depth is an unobserved quantity, if there is not enough observability and baseline, then depth estimates of the 3-D points may not be accurate and therefore fitting a plane leads to poorer performance than estimating an underlying plane modeling the structure. While they assume that the camera positions are provided, we estimate them from visual information and noisy EMT measurements.

The state-of-the-art in terms of drift-free alignment is the well-known bundle adjustment.^14^^,^^15^ This is a batch nonlinear optimization that minimizes the reprojection residuals in all images. As an example close to our application, Atasoy et al.^27^ proposed a vision-based version of bundle adjustment for fibroscopic video mosaicking that weights the images with the number of matched features found in each pair. In Sec. 3, we introduce a problem-specific version of bundle adjustment that fuses the visual and EMT data.

Mur-Artal et al.^19^ showed that it is possible to meet real-time performance requirements with the use of a windowed iterative approach also known as local bundle adjustment (LBA). Under the assumption that the global minimum is too expensive to reach and that the information is provided in an incremental fashion, this algorithm computes the estimates in a window with the underlying idea that cameras outside the window provide little information about the current estimates. Therefore, the estimated cameras are considered fixed in the following iteration and only new cameras are to be estimated. This yields accurate estimates, usually close to the bundle adjustment.^28^ Consequently, we use it in our application to achieve sequential yet accurate mosaics.

The use of an external sensor in our framework aims to constrain the global pose estimates in order to bound the drift. Similar to ours is the work of Agrawal and Konolige,^29^ who explored the idea of using an inexpensive global positioning system combined with a stereo vision system. They used a Kalman filter limiting the drift in translation for long robot trajectories. In our scenario, the fetoscope is close to the tissue and its movement follows a hand-held pattern, which implies additional complications.

Vyas et al.^12^ also integrated an EMT system in a mosaicking pipeline. In their system, the camera movement is restricted to frontoparallel motion. The registration consists of two steps: first, the images are placed according to the EMT measurements and then, a pairwise adjustment is performed using a cross-correlation. This method does make optimal use of the available information since it only enforces pairwise consistency, and the optimization does not take into account the electromagnetic data. By contrast, our probabilistic integration leverages the fact that the EMT measurements are centered in the true camera position and uses jointly either all or a larger subset of information available.

There has been extensive work on the fusion of other types of sensors. The integration of gyrometers,^30^ accelerometers, and inertial measurement units (IMU)^31^ with visual data has been shown to achieve better accuracy and robustness in the estimation of homographies. Additionally, in Ref. 31, an IMU combined with visual keypoints is integrated in a fully probabilistic manner in order to improve the robustness and accuracy of the estimation in a SLAM system. Another idea exploited by some authors is to use predictions of the camera poses and the inertial measurements to recalibrate the bias term^32^^–^^34^ inherent in inertial measurement systems in order to eliminate the drift. To our knowledge, it is not currently possible to fit an inertial system into a clinical fetoscope due to its dimensions.

In our previous work,^7^ we proposed a preliminary model to reduce the drift using the EMT and visual information jointly. In the current work, we extend our framework and validate the complete elimination of the drift.

## 3 Methods

First, we present preliminaries in mosaicking in Sec. 3.1. Then, Sec. 3.2 introduces the EMT measurements and the relationships that used to incorporate them into the mosaicking pipeline. In Sec. 3.3, we detail our main contribution; the formulation of a probabilistic model that achieves a sequential drift-free estimate of the mosaic independently of the number of frames using the complementarity between visual and EMT information. We then state the assumptions of the model and explain the parametrization used. Finally, we introduce the two proposed algorithms to do inference in Sec. 3.4.

### 3.1 Preliminaries in Mosaicking

Given a sequence of 2-D images $I=\{I_{k}{\}}_{k=1}^{N}$ of a planar scene acquired by a hand-held monocular camera with limited field-of-view, where $\pi \in R^{3}$ denotes the plane, we seek to find a representation of the scene, the mosaic $M:\Omega_{M}\to R^{3}$, where $\Omega_{M}\subset R^{2}$ that captures the entire observed area into a 2-D space, where each coordinate is associated with the RGB components of a pixel in the mosaic.

Provided that the structure is a plane, a homography $h(\cdot ):R^{2}\to R^{2}$, which can also be expressed as a matrix $H\in R^{3\times 3}$ in homogeneous coordinates, maps the location of any point $p={\left[\begin{array}{cc} p_{x} & p_{y} \end{array}\right]}^{T}$ in an image to its corresponding point $p^{\prime }={\left[\begin{array}{cc} p_{x}^{\prime } & p_{y}^{\prime } \end{array}\right]}^{T}$ in a second image:

$$p_{x}^{\prime }=\frac{H_{1,1}p_{x},+H_{1,2}p_{y}+H_{1,3}}{H_{3,1}p_{x},+H_{3,2}p_{y},+H_{3,3}},\;p_{y}^{\prime }=\frac{H_{2,1}p_{x}+H_{2,2}p_{y}+H_{2,3}}{H_{3,1}p_{x}+H_{3,2}p_{y}+H_{3,3}}.$$ (1)

If expressed as a matrix, Eq. (1) can be written as ${\tilde{p}}^{\prime }\propto H\tilde{p}$, where H is known up to a scale factor.^35^ We use a tilde in an image point to indicate homogeneous coordinates.

We can relate image k to image j of the sequence with a chain of homographies as follows:

$$H_{j,k}=\overset{j-1}{\underset{l=k}{\prod }}H_{l+1,l},$$ (2)

where the product operator denotes the left matrix multiplication. To build a mosaic, we need to define the common space $\Omega_{M}$, where all images are stitched. Without loss of generality, if we choose the space of the first image as the mosaic space, then Eq. (2) with k=1 expresses the relation between any image j in the sequence and the mosaic space. Throughout this manuscript, a homography with only one subindex relates an image j to the mosaic space, e.g., $H_{j}=H_{j,1}$, whereas a homography with two subindexes relates two images, e.g., $H_{j,k}=H_{j}H_{k}^{-1}$ relates image k to image j.

A pairwise homography $H_{j,k}$ can be directly obtained if a part of the scene is present in both images. In this work, we use a landmark-based approach^36^^–^^38^ to find correspondences in the images. Once the correspondences are computed, an approximation of the homography can be estimated using the DLT algorithm and further refined through a nonlinear optimization.^39^ However, the estimation of a homography between two images inevitably carries error, which leads to accumulation of error when propagated through the chain in Eq. (2). We propose to tackle this problem by incorporating measurements given from an EMT system.

### 3.2 Incorporation of the Electromagnetic Tracker System

**Fig. 2:**
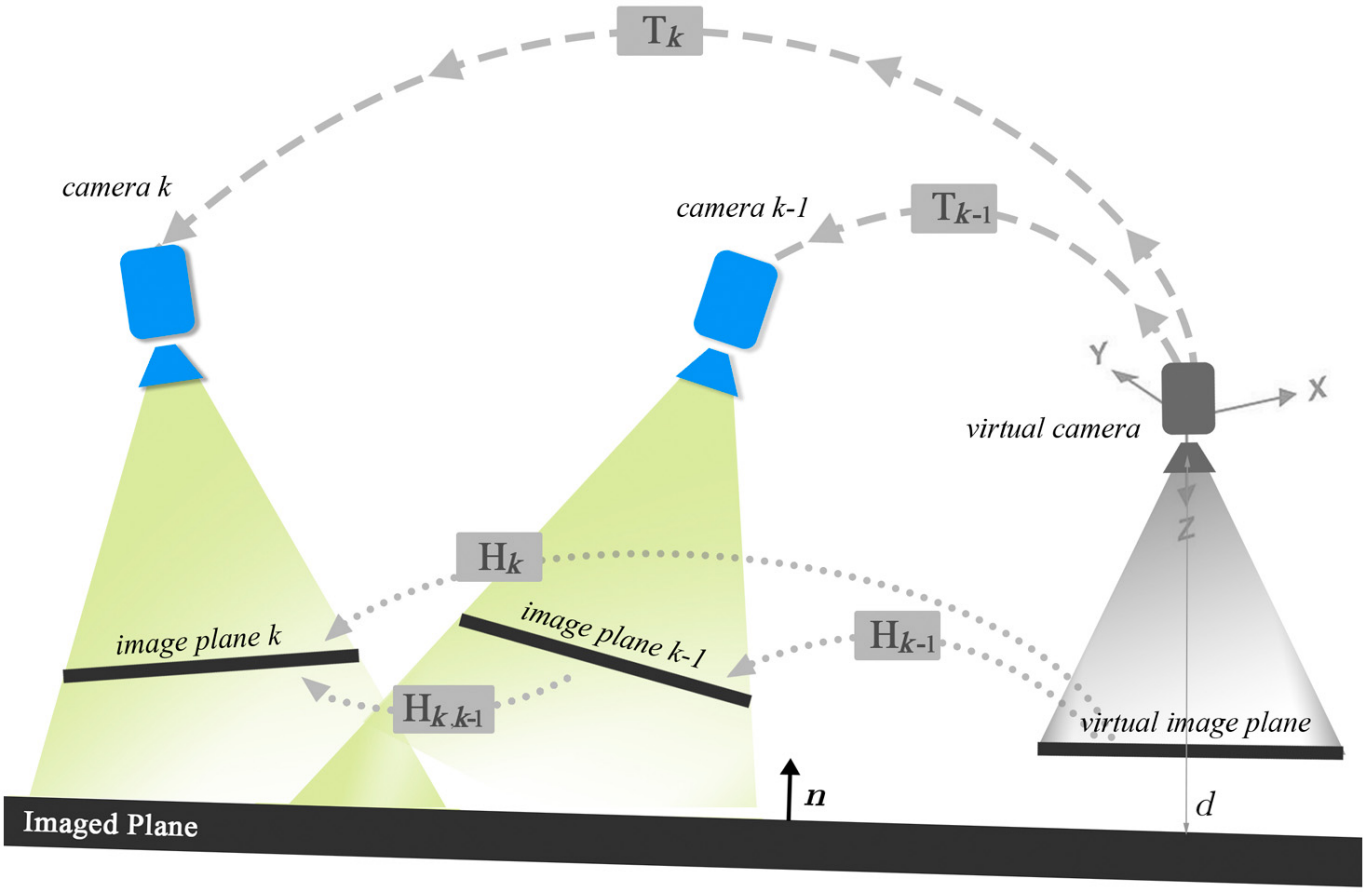
A virtual camera is placed at the origin of an arbitrary global coordinate system. The relation between images and cameras is defined in Eqs. (3) and (5).

We propose to bound the drift in the mosaic by relying on a set of camera pose measurements $Z={\left\{z_{k}\right\}}_{k=1}^{N}$ provided by the EMT system. In order to use EMT measurements in conjunction with visual information, we must establish a link between them. To this end, we place a virtual camera at the origin of an arbitrary global coordinate system, whose image plane can be set to coincide with the mosaic space $\Omega_{M}$. Then, the image plane from the camera pose at time k is related to the image plane of the virtual camera by its homography $H_{k}$, as illustrated in Fig. 2.

Therefore, we can establish the relation as follows:

$$H_{k,k-1}=H_{k}H_{k-1}^{-1}.$$ (3)

Moreover, a 3-D point $p_{3D}$ on the imaged plane satisfies $[\begin{array}{cc} n^{T} & d \end{array}]\left[\begin{array}{c} p_{3D}\\ 1 \end{array}\right]=0$, where the unit vector $n={\left[\begin{array}{ccc} n_{x} & n_{y} & n_{z} \end{array}\right]}^{T}$ and the distance d from the virtual camera to the plane are seen from the point of view of the virtual camera. Let T∈SE(3) be a camera pose expressed as rigid body transformation in 3-D space:

$$T=[\begin{array}{cc} R & t\\ 0 & 1 \end{array}],$$ (4)

where R is a rotation matrix in SO(3) and t is a translation vector in $R^{3}$. Provided that we want to incorporate camera pose measurements, we establish the link between the true camera poses ($T_{k},T_{k-1}$) that induce the homographies ($H_{k},H_{k-1}$) through the plane^24^^,^^35^ as follows:

$$H_{k}=K(R_{k}-t_{k}\frac{n^{T}}{d})K^{-1},$$ (5)

where K is the precalibrated intrinsic camera matrix.

If the plane π was known, then we could compose a mosaic only using the EMT measurements and the images. However, the EMT noise in the camera pose measurements propagates to the mosaic space causing a jitter effect that translates into misregistrations in the composition. More importantly, we do not know π a priori. Therefore, while the guidance of the EMT system is of crucial importance for global positioning consistency, it is necessary to combine both EMT and visual information to integrate knowledge of the scene, implicitly estimate the plane, and obtain pairwise registrations that are as accurate as possible. To this end, we propose a generative probabilistic model that seeks the set of camera poses $X={\left\{x_{k}\right\}}_{k=1}^{N}$ and plane π that generated the sequence of images I and the EMT measurements Z, which are then used to project the images to the mosaic space $\Omega_{M}$ and create the mosaic M:

(X^,π^)=argmax(X,π) P(X,π|Z,I). (6)

We now detail the notation, assumptions, and parametrization used in the probabilistic model.

### 3.3 Probabilistic Model

#### 3.3.1 Notation and modeling assumptions

**Fig. 3:**
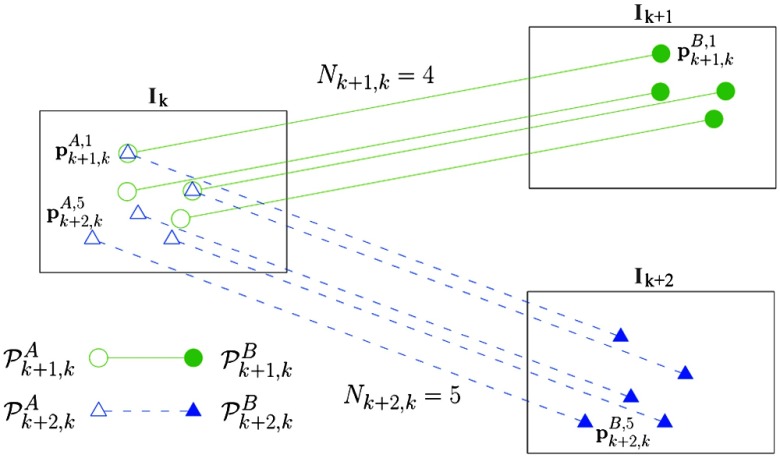
Schematic of the nomenclature of the correspondences. $P_{k+1,k}^{A}$ (green empty circles) is the set of points of image A for which $N_{k+1,k}=4$ correspondences from image k to k+1 are available, being $P_{k+1,k}^{B}$ (green colored circles) the corresponding landmarks in image B. $P_{k+2,k}^{A}$ (blue empty triangles) is the set of points of image A for which $N_{k+2,k}=5$ correspondences from image k to k+2 are available, being $P_{k+2,k}^{B}$ (blue colored triangles) the corresponding landmarks in image B. As an example, the landmark $p_{k+1,k}^{A,1}$ corresponds to the point $p_{k+1,k}^{B,1}$ and the point $p_{k+2,k}^{A,5}$ corresponds to the point $p_{k+2,k}^{B,5}$.

Consider two images. Let image A be the source image and image B be the target image. These images each contain a set $P_{l,m}^{A}$ and $P_{l,m}^{B}$ of $N_{l,m}$ corresponding landmarks found from image m to image l. For each set, let us define the i'th corresponding landmark in the set as $\left\{p_{l,m}^{A,i}\in R^{2}|P_{l,m}^{A}={\left\{p_{l,m}^{A,i}\right\}}_{i=1}^{N_{l,m}}\right\}$ for image A and $\left\{p_{l,m}^{B,i}\in R^{2}|P_{l,m}^{B}={\left\{p_{l,m}^{B,i}\right\}}_{i=1}^{N_{l,m}}\right\}$ for image B. For simplicity, we assume that landmarks in an image are independent. Additionally, for different pairs of images, we consider independence of all the sets in source images in $P^{A}=\underset{l,m\in L}{⋃}P_{l,m}^{A}$, and target images $P^{B}=\underset{l,m\in L}{⋃}P_{l,m}^{B}$, where L is the set of all possible corresponding image indexes. Figure 3 depicts a schematic of the nomenclature of the correspondences.

In terms of the parametrization, we use a scaled normal vector $\pi =n/d\in R^{3}$ to parametrize the plane. Compared to other parametrizations,^23^ this has the advantage of encoding the inverse depth (1/d) in each of the components, reducing the nonlinearity and thus accelerating the convergence. We use a minimal parametrization of six parameters for the camera poses $x_{k}={\left[\begin{array}{cc} r_{k}^{T} & t_{k}^{T} \end{array}\right]}^{T}$ with the orientation $r={\left[\begin{array}{ccc} r_{x} & r_{y} & r_{z} \end{array}\right]}^{T}$ being the Euclidean vector in $R^{3}$ identified with the skew-symmetric matrix S∈so(3) for which $R=e^{S}\in \mathrm{SO}(3)$:

$$S=[\begin{array}{ccc} 0 & -r_{z} & r_{y}\\ r_{z} & 0 & -r_{x}\\ -r_{y} & r_{x} & 0 \end{array}].$$ (7)

This parametrization ensures valid rotation matrices and is valid for all angles as the exponential map in so(3) is subjective. Furthermore, the camera is guaranteed to look downward, toward the placenta, and therefore, the exponential map is bijective for all angles of interest as long as the camera does not complete a full rotation around its z axis, which is very unlikely.

Once the notation has been defined, we state the main modeling assumptions:

1. We consider the imaged object to be a plane.^7^
2. Every EMT measurement $z_{k}$ is modeled as a Gaussian random variable centered on the true camera pose $x_{k}$ with diagonal covariance $\Sigma_{\mathrm{EMT}}$, that is $z_{k}|x_{k}∼N_{z_{k}}(x_{k},\Sigma_{\mathrm{EMT}})$. Even though the EMT measurements are actually not strictly Gaussian,^40^^,^^41^ this is a common assumption^40^ that simplifies the problem. We account for modeling errors in the EMT measurements by enlarging its standard deviation.
3. The locations of corresponding points between adjacent images match the same visual content, but they are imperfect; each pair of corresponding points has a matching error defined as the distance between a point in an image and its correspondence propagated from the other image. For simplicity, we assume this error to have zero mean and diagonal covariance matrix $\sigma_{v}^{2}I$. This is equivalent to saying that a 2-D point in image B is a Gaussian measurement generated from a true 2-D point in image A, which is $p_{l,m}^{B,i}|x_{l},x_{m},\pi ,p_{l,m}^{A,i}∼N_{p_{l,m}^{B,i}}(\mu_{v}^{i},\sigma_{v}^{2}I)$ in which the mean $\mu_{v}^{i}(x_{l},x_{m},\pi ,p_{l,m}^{A,i})$, detailed later in Eq. (13), is the projected location of the point in image A. See Sec. 5 for more comments about this modeling assumption.
4. We assume that the camera is moving smoothly and, therefore, model the relation between camera poses with a constant velocity motion model^42^ with mean $\mu_{p}(x_{k-1},x_{k-2})$, which is described later in Eq. (15), and diagonal covariance matrix $\Sigma_{p}$, that is $x_{k}|x_{k-1},x_{k-2}∼N_{x_{k}}(\mu_{p},\Sigma_{p})$. This model expresses that the velocity at time k must be the same as the velocity at time k−1 plus a perturbation.

Within this probabilistic framework, the estimation of the mosaic can be cast as a Bayesian inference problem, in which the posterior $P(X,\pi |Z,P^{A},P^{B})$ is maximized with respect to the camera poses X and plane π:

(X^,π^)=argmax(X,π) P(X,π|Z,PA,PB) (8)

in which the posterior probability factorizes as follows:

$$P(X,\pi |Z,P^{A},P^{B})\propto P(Z,P^{B}|X,\pi ,P^{A})P(X,\pi |P^{A})\;\propto \underset{\left(a\right)}{\underset{⏟}{P(Z|X)}}\underset{\left(b\right)}{\underset{⏟}{P(P^{B}|X,\pi ,P^{A})}}\underset{\left(c\right)}{\underset{⏟}{P(X)P(\pi )}}.$$ (9)

First, we applied Bayes theorem and dropped the constant factor. Second, if the true cameras X are given, then the EMT measurements Z are independent of the visual terms $P^{B}$ and therefore, $P(Z,P^{B}|X,\pi ,P^{A})$ can be separated as (a) and (b). Furthermore, in (a), we applied the fact that the EMT measurements are independent of the plane π given X so that P(Z|X,π)=P(Z|X).

**Fig. 4:**
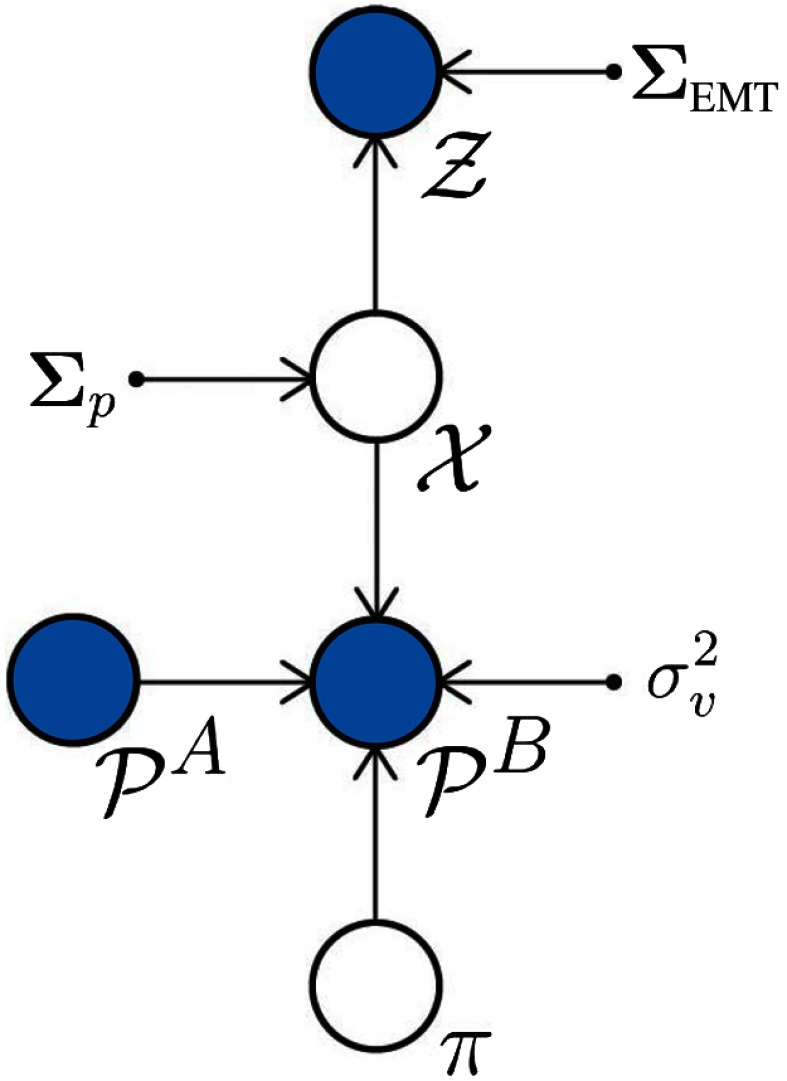
Graphical model of the proposed probabilistic framework. Circles represent random variables, which can be either latent (white background) or observed (shaded). Parameters of the model are depicted as a point.

In the prior term identified as (c), we have first considered independence of $P^{A}$ and π and then independence of π and X, i.e., $P(X,\pi |P^{A})=P(X,\pi )=P(X)P(\pi )$. We assume that we do not have prior information about the plane and that its distribution is bounded, thus considering P(π)∝1. The graphical model of the proposed probabilistic framework is presented in Fig. 4. Circles represent random variables, which can be either latent (white background) or observed (shaded). Parameters of the model are depicted as a point.

Next, we incorporate the assumptions and present the factorization of the likelihood and prior terms.

#### 3.3.2 Likelihood and prior

The likelihood combines the EMT term P(Z|X) and visual term $P(P^{B}|X,\pi ,P^{A})$. In particular, the EMT term contains the relation between the EMT measurements and the true cameras, assuming independence of the EMT measurements, can be expressed as follows:

$$P(Z|X)=\prod_{k=1}^{N}P(z_{k}|x_{k}).$$ (10)

The visual term comes from the correspondences between images, and it enforces visual pairwise consistency. Applying the assumption that sets of corresponding points are independent of each other, we can simplify this term by factorizing it as follows:

$$P(P^{B}|X,\pi ,P^{A})=\prod_{\{m,l\}\in L}P(P_{l,m}^{B}|x_{l},x_{m},\pi ,P_{l,m}^{A}).$$ (11)

In every image, we also assume every landmark to be independent. Therefore, we can further decouple the points as follows:

$$P(P_{l,m}^{B}|x_{l},x_{m},\pi ,P_{l,m}^{A})=\prod_{i=1}^{M^{l,m}}P(p_{l,m}^{B,i}|x_{l},x_{m},\pi ,p_{l,m}^{A,i}).$$ (12)

We assume every point in image B to be $p_{l,m}^{B,i}|x_{l},x_{m},\pi ,p_{l,m}^{A,i}∼N_{p_{l,m}^{B,i}}(\mu_{v}^{i},\sigma_{v}^{2}I)$ with

$$[\begin{array}{c} \mu_{v}^{i}(x_{l},x_{m},\pi ,p_{l,m}^{A,i})\\ 1 \end{array}]\propto H_{l}H_{m}^{-1}{\overset{˜}{p}}_{l,m}^{A,i}=(R_{l}-t_{l}\frac{n^{T}}{d}){\left(R_{m}-t_{m}\frac{n^{T}}{d}\right)}^{-1}{\overset{˜}{p}}_{l,m}^{A,i}$$ (13)

For convenience, we redefine every point $\tilde{p}$ directly in the normalized image space through $\tilde{p}=K^{-1}\tilde{q}$ being $\tilde{q}$ a homogeneous point in the image space.

The number of correspondences has a strong impact on the estimation. A high number emphasizes the visual term, unbalancing the fusion with the EMT information. This effect is related to the simplifying independence assumption between landmarks. The solution adopted has been to normalize and manually adapt the visual variance $\sigma_{v}^{2}$ to ensure good interframe registrations, which can be seen as a pragmatic correction factor. A more detailed explanation of this problem is covered in Sec. 5.

The prior term P(X) on the camera poses is approximated by using a second order Markov process, which accounts for a constant velocity of the camera. We assume no prior knowledge of the joint probability on a bounded region of the space, i.e., $P(x_{2},x_{1})\propto 1$:

$$P(X)=P(x_{1},\ldots ,x_{N})=P(x_{2},x_{1})\overset{N}{\underset{k=3}{\prod }}P(x_{k}|x_{k-1},x_{k-2},\ldots ,x_{1})\;\approx P(x_{2},x_{1})\overset{N}{\underset{k=3}{\prod }}P(x_{k}|x_{k-1},x_{k-2})\propto \overset{N}{\underset{k=3}{\prod }}P(x_{k}|x_{k-1},x_{k-2}).$$ (14)

We assume the new camera motion $x_{k}|x_{k-1},x_{k-2}∼N_{x_{k}}(\mu_{p},\Sigma_{p})$, where

$$\mu_{p}(x_{k-1},x_{k-2})={\left[\begin{array}{cc} {\overset{¯}{r}}_{k}^{T} & {\overset{¯}{t}}_{k}^{T} \end{array}\right]}^{T}$$ (15)

is decomposed from the rigid transformation ${\bar{T}}_{k}$ into rotation and translation:

$${\overset{¯}{T}}_{k}=[\begin{array}{cc} {\overset{¯}{R}}_{k} & {\overset{¯}{t}}_{k}\\ 0 & 1 \end{array}]=T_{k-1,k-2}T_{k-1},$$ (16)

with ${\bar{R}}_{k}=\exp({\bar{r}}_{k})$. This simply says that the last camera pose $T_{k-1}$ is composed with the last available pairwise velocity $T_{k-1,k-2}=T_{k-1}T_{k-2}^{-1}$, giving an approximate idea of where the current estimate should be.

### 3.4 Inference

By applying a negative logarithm to the posterior probability distribution, we can express the proposed model as the minimization of a cost, which contains three terms, the visual cost $C_{v}$, the EMT cost $C_{\mathrm{EMT}}$, and the cost associated with the temporal model $C_{p}$ as follows:

(X^,π^)=argmin(X,π) (Cv+CEMT+Cp), (17)

where

$$C_{v}=\underset{l,m\in L}{\sum }\overset{N_{l,m}}{\underset{i=1}{\sum }}\frac{1}{\sigma_{v}^{2}}{\left\|p^{B,i}-\mu_{v}^{i}(x_{l},x_{m},\pi ,p_{l,m}^{A,i})\right\|}_{2}^{2},$$ (18)

$$C_{\mathrm{EMT}}=\overset{N}{\underset{k=1}{\sum }}{\left(z_{k}-x_{k}\right)}^{T}\Sigma_{\mathrm{EMT}}^{-1}(z_{k}-x_{k}),$$ (19)

$$C_{p}=\overset{N}{\underset{k=3}{\sum }}{\left[x_{k}-\mu_{p}(x_{k-1},x_{k-2})\right]}^{T}\Sigma_{p}^{-1}[x_{k}-\mu_{p}(x_{k-1},x_{k-2})].$$ (20)

This is a large scale nonlinear least squares problem, which can be solved using a Gauss–Newton method, for which the EMT measurements can be used for initialization.

If only the visual cost is used, the problem results in bundle adjustment. Therefore, the proposed algorithm is an adapted version of bundle adjustment that also incorporates the EMT measurements and temporal consistency of the camera motions. These algorithms require to have all the information beforehand, i.e., they are offline. This is prohibitive in our case since we aim for a sequential estimate. Consequently, we move toward local methods. However, we do consider the incorporation of the EMT information in bundle adjustment and propose it as an additional contribution, since it can be used for refinement at the end of the scanning procedure and can serve as a reference.

We use LBA, an approximation of bundle adjustment in which only the components within a temporal window of size W are considered. This drastically reduces the computational burden of the algorithm and allows for sequential estimation. We slightly modify this approach following these two main assumptions: (i) The cameras far from the current window provide little information about the new cameras to be estimated, yet they provide information about the plane given visual measurements. (ii) The cameras that have already been estimated are considered fixed in the next iteration. These assumptions are further commented in Sec. 5.

Let $X_{e}=\{x_{k}^{e}{\}}_{k=1}^{\chi_{e}}$ be the subset of cameras to be estimated, where $\chi_{e}$ is the number of cameras in the subset, $X_{g}=\{x_{k}^{g}{\}}_{k=1}^{\chi_{g}}$ is the set of cameras already estimated and fixed within the window, with $\chi_{g}$ being the number of cameras in the subset as well as the index of the most recent fixed camera, such that $W=\chi_{e}+\chi_{g}$. Additionally, let $X_{o}$ be the subset of cameras already estimated outside the window, such that $X=\{X_{e},X_{g},X_{o}\}$. Analogously, let $Z_{e}$ be the set of EMT measurements corresponding to the camera poses to be estimated.

We now seek to maximize the posterior probability of the new camera motions $X_{e}$ and plane π given all the estimated cameras that have been fixed, and the EMT and visual measurements such that

(X^e,π^)=argmax(Xe,π) P(Xe,π|Xg,Xo,Ze,PA,PB). (21)

In this case, the posterior factorizes as follows:

$$P(X_{e},\pi |X_{g},X_{o},Z_{e},P^{A},P^{B})\propto P(Z_{e},P^{B}|X,\pi ,P^{A})P(X_{e},\pi |X_{g},X_{o},P^{A})\;\propto P(Z_{e}|X)P(P^{B}|X,\pi ,P^{A})P(X_{e}|X_{g},X_{o})P(\pi ).$$ (22)

Provided that $Z_{e}$ are independent of the rest of the camera poses given $X_{e}$, then $P(Z_{e}|X)\propto P(Z_{e}|X_{e})$. Additionally, we approximate $P(X_{e}|X_{g},X_{o})$ with the assumption that the cameras outside the temporal window do not influence the estimation of the new ones as follows:

$$P(X_{e}|X_{g},X_{o})\approx P(X_{e}|X_{g})\approx P(x_{2}^{e}|x_{1}^{e},x_{\chi_{g}}^{g})P(x_{1}^{e}|x_{\chi_{g}}^{g},x_{\chi_{g}-1}^{g})\times \overset{\chi_{e}-3}{\underset{k=0}{\prod }}P(x_{\chi_{e}-k}^{e}|x_{\chi_{e}-(k+1)}^{e},x_{\chi_{e}-(k+2)}^{e}),$$ (23)

where all the terms have been further approximated as a Markov process of second order in the same way as before.

**Fig. 5:**
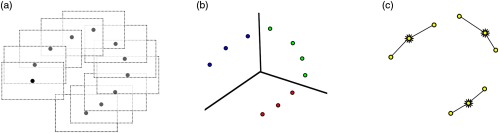
(a) The estimated cameras and plane produce drift-free estimates of the projected images in the mosaic space. We project the image corners (dotted contour) and compute the centroids (black dots). (b) The centroids have been clustered using K-means. (c) A consecutive subset of centroids has been randomly selected from each cluster. These centroids correspond to fixed camera poses as well as visual measurements, which are then used to leverage the visual pairwise relations in different areas of the space.

However, operating only in a temporal window may not provide enough baseline between camera poses to capture the depth of the plane accurately. Therefore, we have enhanced the measurement set with evenly distributed visual measurements that cover the available space explored at every iteration. In order to sparsely select sets of landmarks throughout the observed area, we project the image corners using the available camera poses and plane estimated at a given iteration. Provided that drift has not been accumulated during the estimation, we can use the projection of the image corners to determine the location of the image in the mosaic space. To this end, we compute the centroids of the reprojected corners and use K-means [Fig. 5(b)] to cluster the centroids into different regions of the space. Finally, we randomly pick a consecutive subset of camera motions in each cluster to be taken into account together with their corresponding landmarks. Figure 5 depicts the proposed algorithm.

In a similar way as before, we can formulate the problem:

(X^e,π^)=argmin(Xe,π) (Cv+CEMT+Cp), (24)

where

$$C_{v}=\underset{l,m\in W}{\sum }\overset{N_{l,m}}{\underset{i=1}{\sum }}\frac{1}{\sigma_{v}^{2}}{\left\|p^{B,i}-\mu_{v}^{i}(x_{l},x_{m},\pi ,p_{l,m}^{A,i})\right\|}_{2}^{2},$$ (25)

$$C_{\mathrm{EMT}}=\overset{W}{\underset{k=1}{\sum }}{\left(z_{e,k}-x_{k}^{e}\right)}^{T}\Sigma_{\mathrm{EMT}}^{-1}(z_{e,k}-x_{k}^{e}),$$ (26)

$$C_{p}=\overset{W}{\underset{k=3}{\sum }}{\left[x_{k}^{e}-\mu_{p}(x_{k-1}^{e},x_{k-2}^{e})\right]}^{T}\Sigma_{p}^{-1}[x_{k}^{e}-\mu_{p}(x_{k-1}^{e},x_{k-2}^{e})]\;+{\left[x_{2}^{e}-\mu_{p}(x_{1}^{e},x_{\chi_{g}}^{g})\right]}^{T}\Sigma_{p}^{-1}[x_{2}^{e}-\mu_{p}(x_{1}^{e},x_{\chi_{g}}^{g})]\;+{\left[x_{1}^{e}-\mu_{p}(x_{\chi_{g}}^{g},x_{\chi_{g}-1}^{g})\right]}^{T}\Sigma_{p}^{-1}[x_{1}^{e}-\mu_{p}(x_{\chi_{g}}^{g},x_{\chi_{g}-1}^{g})],$$ (27)

where W⊂L is the subset of corresponding images that has been sparsely selected as well as the ones within the temporal window. This results in much smaller nonlinear least square problems that can be solved using Gauss–Newton.

LBA produces a slightly different version of the plane in every iteration. The task of composing consistent homographies from these estimates is not trivial. For simplicity, we have opted to compose the new homographies with the estimated set of cameras and plane obtained in every iteration by using Eq. (5). This may lead to slight misregistrations in the mosaic between estimates with different planes when the camera has not explored enough area. Eventually, the estimate of the plane is going to converge and could potentially be assumed fixed, simplifying the optimization problem.

## 4 Experiments and Results

In this section, we introduce the algorithms to compare to, the datasets, and the metrics used to then present a suite of experiments, where we prove that our approach is drift-free, robust to EMT noise as well as robust to loss of visual information.

### 4.1 Algorithms, Datasets, and Evaluation Metric

#### 4.1.1 Algorithms

We name our proposed algorithm LBAVis+EMT. We compare it against the pairwise solution (PairVis) of the mosaicking pipeline that Brown et al.^14^ proposed as initialization for a further global refinement step. We also compare it against the algorithm established as the state-of-the-art in global alignment, so-called bundle adjustment^16^ (BAVis). However, rather than using homographies to parametrize the problem, we used the same parametrization as in LBAVis+EMT to avoid over-parametrization. We also compare LBAVis+EMT against the proposed version (BAVis+EMT) of bundle adjustment that incorporates the EMT information.

#### 4.1.2 Datasets

**Fig. 6:**
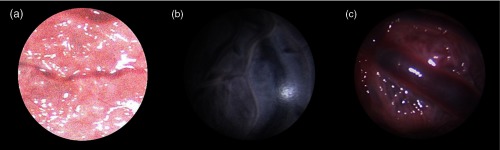
(a) The synthetic (SYN), (b) phantom-based (PHB), and (c) *ex vivo* human placenta (EX) datasets.

We introduce a synthetic (SYN, 3370 frames), a phantom-based (PHB, 902 frames), and an *ex vivo* human placenta (EX, 366 frames) datasets, which are composed of a set of EMT measurements as well as a sequence of images from which correspondences have been obtained using SIFT and RANSAC.^14^ Experimental procedures for the acquisition of the *ex vivo* human placenta were approved by Bloomsbury National Research Ethics Service Committee and by University College London Hospital Research and Development (REC Reference number 133888). The SYN is a xy-translation synthetic dataset in which the camera motion follows a circular pattern. It contains EMT information synthetically generated following the assumptions made on the EMT and visual information (see steps 2 and 3). The sequence of images was generated by selecting image regions (368×378) of a large image, representing the imaged plane, observed by the ground truth cameras. The PHB (783×782) and EX (806×779) are hand-held datasets. The PHB is recorded by imaging a printed version of a placenta taped onto a planar surface. Example images for all datasets are shown in Fig. 6.

**Fig. 7:**
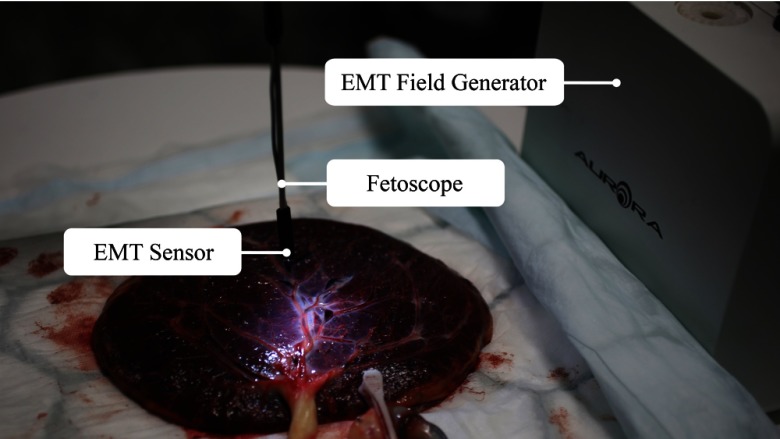
The setup is composed of an EMT field generator and fetoscope in which an EMT sensor has been assembled at its tip.

The PHB and EX datasets were recorded using the following setup: a camera head IMAGE1 H3-Z SPIES mounted on a 3-mm straight scope 26007 AA 0 (Karl Storz Endoskope, Tuttlingen, Germany), an EMT system NDI Aurora with a planar field generator and a Mini 6 DoF sensor. According to Franz et al.,^9^ the MSE in the accuracy of the system is 0.9 deg in the rotation and 0.25 mm in the translation in laboratory conditions. However, since the accuracy of the EMT system can vary due to external factors, such as metal in the working area or position in the working volume, dynamic electromagnetic tracking errors, synchronization errors, and hand-eye calibration errors, we arbitrarily take larger^9^ standard deviations of 1 deg and 1 mm as default values in our experiments. Synchronized video (25 fps) and EMT data (40 Hz) were obtained using the NifTK^43^ software with a maximum synchronization error of 12.5 ms. The fetoscope was precalibrated using the MATLAB camera calibration toolbox in Ref. 44. We also precomputed and applied the hand-eye calibration^45^ matrix from a sequence of images of a checkerboard as well as synchronized sensor poses. The reference plane was obtained by fitting a plane to a large sweep of 3-D points collected by scanning the surface with an EMT sensor. We obtained the ground truth homographies by manually registering the fetoscopic images directly to the original image of the placenta. The entire setup is shown in Fig. 7.

To provide quantitative results in terms of the accuracy of a mosaic with respect to the ground truth, we need to define the metrics.

#### 4.1.3 Metrics

We parametrize a mosaic as a collection of homographies. Therefore, comparing two mosaics becomes equivalent to comparing two collections of homographies. Starting by comparing individual homographies, we define the error between any homography H and the ground truth homography $H_{GT}$ as the mean residual error of a projected grid of points $\{\rho_{i}{\}}_{i=1}^{N_{g}}\in \Omega_{I}$ from the image space $\Omega_{I}\subset R^{2}$ to the mosaic space $\Omega_{M}$:

$$e_{j}=\frac{1}{N_{g}}\overset{N_{g}}{\underset{i=1}{\sum }}\|w(H_{j}^{-1},\rho_{i})-w(H_{j,\;GT}^{-1},\rho_{i}){\|}_{2},$$ (28)

where $w(H_{j},\rho )$ projects the point ρ from the image space $\Omega_{I}$ to the mosaic space $\Omega_{M}$ through $H_{j}$ by propagating the point and converting it to Cartesian coordinates. Specifically, we have used a grid of $N_{g}=100^{2}$ points for each comparison.

To further compare two collections of homographies, we take the mean of the error associated to each of the homographies with respect to the ground truth and define the error $e_{M}$ that represents the average reprojection error in pixels:

$$e_{M}=\frac{1}{N}\sum_{j=1}^{N}e_{j}.$$ (29)

### 4.2 Experimental Suite

In the first experiment, we show that LBAVis+EMT is not affected by long-term drift in the SYN and PHB datasets. In the second experiment, we demonstrate that the complementarity between EMT and visual information makes the system robust to the jitter effect caused by the EMT noise on the SYN dataset. In the third experiment, we prove how the loss of visual information does not impede the creation of the mosaic in the SYN dataset. Finally, we present a set of videos for all datasets showing accurate sequential drift-free creation of the mosaics from long sequences.

The choice of parameters has been the following: W=5, $\chi_{e}=3$, and K=3, each of which we take 5 consecutive cameras for SYN; W=3, $\chi_{e}=1$, and K=3 each of which we take 3 consecutive cameras for PHB; and W=12, $\chi_{e}=6$, and K=3 each of which we take 12 consecutive cameras for EX. The covariance matrix $\Sigma_{p}$ has been estimated through an independent dataset with similar motion characteristics. $\Sigma_{\mathrm{EMT}}$ has been conservatively chosen to account for other sources of error while $\sigma_{v}^{2}$ has been set to $\sigma_{v}=1$ pixel. $\Sigma_{\mathrm{EMT}}$ and $\Sigma_{p}$ are shown in Appendix A.

Once the alignment has been performed, we use a linear blending^15^ with a thin circular black border in each image to clearly show where it has been stitched. The use of linear blending allows for clearly distinguishing the misregistrations and it is, therefore, more interesting for demonstration purposes. However, we also use multiband blending^15^ to provide more appealing results for all datasets (Videos 1–3).

#### 4.2.1 Drift-free mosaicking

**Fig. 8:**
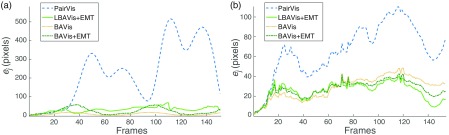
Assessment of the accuracy of the PairVis (blue dashed), LBAVis+EMT (green), BAVis (yellow dotted), and BAVis+EMT (dark green dash-dotted) in (a) SYN and (b) PHB.

The goal of this experiment is to show that LBAVis+EMT does not drift over time. Figure 8 shows the curves for the PairVis (blue dashed), LBAVis+EMT (green), BAVis (yellow dotted), and BAVis+EMT (green dotted) in the SYN [Fig. 8(a)] and PHB [Fig. 8(b)] datasets. The x-axis is the number of frames and the y-axis is the error $e_{j}$ in pixels corresponding to a homography $H_{j}$. Note that in the case of PairVis, this homography is created through a composition of pairwise homographies, and therefore, a point in the curve shows the cumulative error up to the corresponding frame.

Both experiments show a similar trend; the growing tendency in the PairVis is expected due to small misregistrations in subsequent images, which leads to this long-term drift. By contrast, LBAVis+EMT maintains an approximately constant tendency over time, which demonstrates the absence of long-term drift. Additionally, the accuracy of the proposed approach is very close to that of BAVis and BAVis+EMT. This shows the feasibility of sequential methods for mosaicking in a planar scenario in terms of accuracy, when the EMT system is guiding the estimation.

We have used a relatively small number of frames (152 frames in SYN and 155 in PHB) to prove the long-term drift in PairVis while being able to compute BAVis and BAVis+EMT. However, we emphasize that after a certain number of frames, which will depend on the quality of the pairwise registrations, the accumulated error can lead to projections of unnatural size (see Fig. 1), which may result in memory problems when creating the mosaic image. By contrast, we show in Fig. 11, how LBAVis+EMT can cope well with long sequences.

In Table 1, we show the runtimes for both SYN and PHB datasets. We report the runtimes of the steps that differ between algorithms: matching and optimization. The experiments were performed in a MacBook Pro with an Intel Core I7 at 2.5GHz with 4 cores and 16 GB of RAM memory and the algorithms were implemented in MATLAB using VLFeat version of SIFT and matching algorithm.^46^ While PairVis is the fastest method, LBAVis+EMT shows similar performance to the gold standard bundle adjustment with much less computational burden. We also highlight the fact that the computational times of BAVis+EMT are smaller than BAVis. Since we provide direct, albeit noisy measurements of the latent variables to estimate, the problem is better posed. Therefore, faster convergence is expected.

**Table 1:** Runtimes for both SYN and PHB datasets for PairVis, LBAVis+EMT, BAVis, and BAVis+EMT. The second column indicates the number of images in the dataset. The third column corresponds to the number of matched pairs. The fourth column is the average number of correspondences in all pairs. The fifth column is the matching runtime in seconds. Note that it includes unsuccessful matching attempts. The last column is the optimization runtime in seconds.

Algorithm	Dataset	No. of images	No. of pairs	No. Avg. Corr.	Matching (s)	Optimization (s)
PairVis	SYN	152	151	86	49.811	3.145
LBAVis+EMT	SYN	152	350	67	178.766	38.567
BAVis	SYN	152	1094	76	4286.259	521.819
BAVis+EMT	SYN	152	1094	76	4350.518	321.928
PairVis	PHB	155	154	222	88.192	3.333
LBAVis+EMT	PHB	155	449	184	519.447	50.638
BAVis	PHB	155	2030	105	15064.135	1488.251
BAVis+EMT	PHB	155	2030	105	15394.628	1103.748

#### 4.2.2 Robustness to electromagnetic tracker noise

Inherently, the use of EMT information produces a jitter effect in the mosaic due to the noise in the camera pose measurements. The goal of this experiment is to assess the accuracy of LBAVis+EMT and show that when EMT information is fused with visual information, the jitter effect is mitigated in the creation of the mosaic. For this purpose, we created seven synthetic datasets, each with different EMT noise statistics. We have denoted the scalar ν to be the standard deviation of the EMT noise in the camera poses. We chose a small subset of only 17 frames since we are now only interested in the quality of pairwise registrations.

**Fig. 9:**
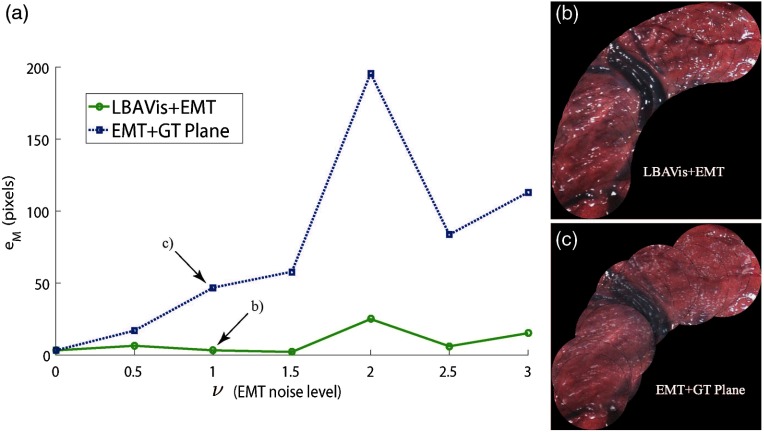
(a) In the x-axis, the multiplier ν defines the standard deviation of the rotation and translation in degrees and millimeters, respectively, of seven versions of the SYN dataset. In the y-axis, the error in pixels $e_{M}$ in the mosaic. Every point in the graph represents the error in a mosaic created by using either LBAVis+EMT (green) or EMT (blue dotted) with the ground truth plane (EMT+GT Plane). (b) LBAVis+EMT mosaic for ν=1. (c) EMT + GT plane mosaic for ν=1.

For every SYN dataset, we assessed the accuracy of the resulting mosaic for LBAVis+EMT and compared it against the jittery baseline composition of the mosaic using only EMT information and the ground truth plane. Figure 9(a) displays the graphs for both algorithms. The x-axis represents the different datasets from best to worse EMT noise statistics ν while the y-axis corresponds to the average error in the mosaic $e_{M}$ measured in pixels.

We can see how while the EMT-based composition shows an approximately linear tendency with the increase of ν, LBAVis+EMT outperforms it by showing an approximately constant accuracy. This stands to reason since, after ν=1, the quality of the EMT information is really bad and thus barely used. Then, the visual information plays a major role in the estimation. We have highlighted the case ν=1 and displayed the mosaics corresponding to ν=1 since it is the value of choice in other experiments. Figure 9(b) shows an accurate mosaic created with LBAVis+EMT, and Fig. 9(c) shows the jitter effect of EMT information in the mosaic.

#### 4.2.3 Robustness to sudden loss of visual information

**Fig. 10:**
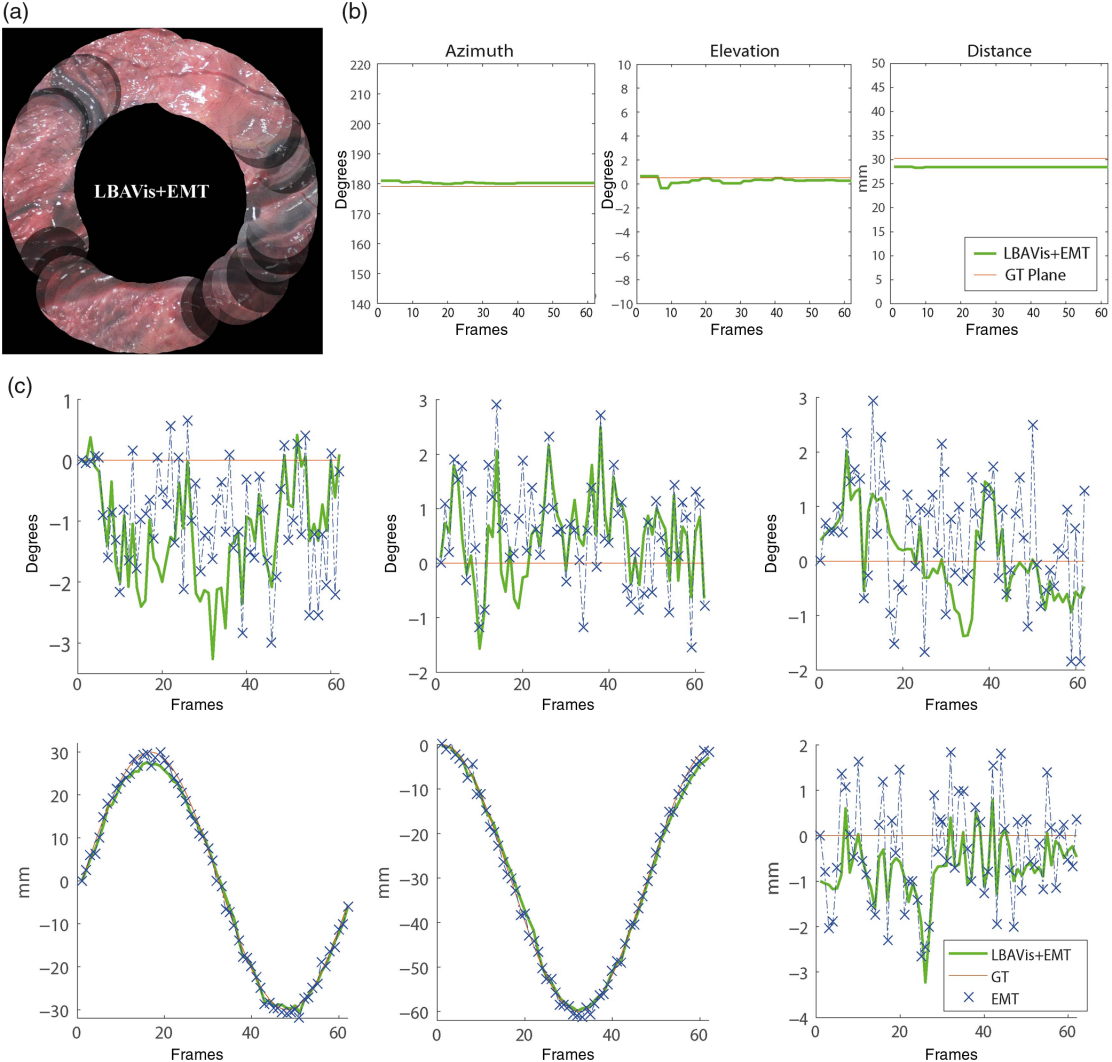
(a) LBAVis+EMT mosaic of a synthetic dataset where some random frames have been replaced by black frames to simulate the lack of visual content (7, 11, 12, 23, 24, 37, 38, 42, 43, 45, 51, and 54). Black circles in the mosaic represent the reprojection of the missing frames in the mosaic space. (b) Estimation of the plane (green) and ground truth (red). (c) Estimation of the camera pose (green); rotation ($r_{x},r_{y},r_{z}$) and translation ($t_{x},t_{y},t_{z}$), EMT measurements (blue crosses), and ground truth (red).

In this experiment, we test our approach when no corresponding landmarks can be found between pairs of frames. We created an alternative dataset in the same manner than the SYN dataset was created. This new dataset contains 62 frames of which 12 randomly selected ones were replaced by black frames to simulate lack of visual content. The black frames are 7, 11, 12, 23, 24, 37, 38, 42, 43, 45, 51, and 54. Then, we ran LBAVis+EMT to assess its accuracy with missing visual information. Figure 10(a) shows how despite loss of visual information, when PairVis would fail, LBAVis+EMT has been able to successfully create a mosaic. Black circles in the mosaic [Fig. 10(a)] represent the reprojection of the missing frames in the mosaic space. Figures 10(b) and 10(c) show the estimated plane and six components of the camera pose (green), rotation ($r_{x},r_{y},r_{z}$) and translation ($t_{x},t_{y},t_{z}$), the EMT measurements (blue crosses) and their ground truth (red), respectively, for each frame. We convert the plane to azimuth, elevation, and distance for an easier interpretation and detail the conversion in Appendix A. Therefore, when no landmarks are available, the estimation can continue successfully and provide a reasonable estimate of the camera poses and plane.

**Fig. 11:**
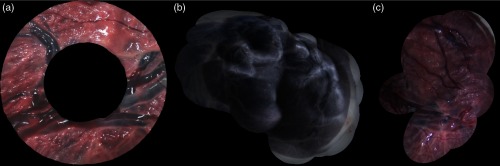
Mosaic using LBAVis+EMT in the (a) SYN (Video 1), (b) PHB (Video 2), and (c) EX (Video 3) datasets. (Video 1, MP4, 5.1MB [URL: https://doi.org/10.1117/1.JMI.5.2.021217.1]; Video 2, MP4, 3.4MB [URL: https://doi.org/10.1117/1.JMI.5.2.021217.2]; and Video 3, MP4, 3.1MB [URL: https://doi.org/10.1117/1.JMI.5.2.021217.3])

#### 4.2.4 Sequential creation and blending of the mosaics

**Fig. 12:**
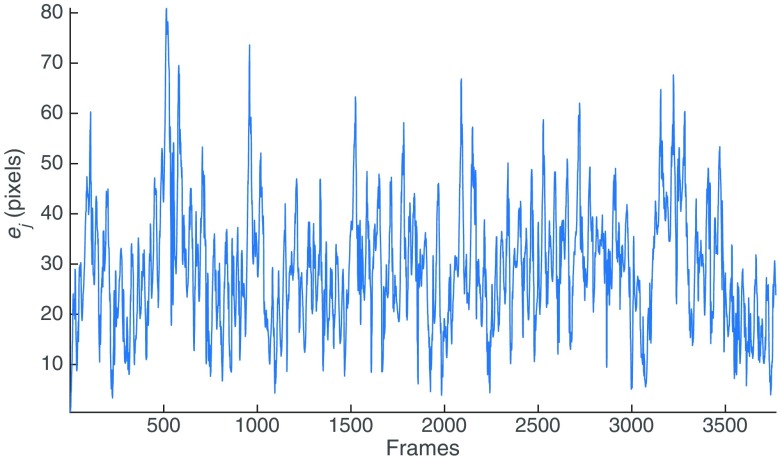
Error $e_{j}$ in pixels of LBAVis+EMT in the SYN dataset.

Figures 11(a)–11(c) show the results of running LBAVis+EMT in the SYN (3770 frames), PHB (902 frames), and EX (366 frames) datasets, respectively. Figure 12 shows the graph corresponding to the error $e_{j}$ in every frame of the SYN dataset. We provide videos (Videos 1–3) that illustrate the sequential creation of the mosaics by showing the subsequent blending of every new image into the mosaic image. We recommend the reader visualize the videos for a better understanding of the results. In this experiment, the goal is to demonstrate that our approach can create accurate long mosaics in a sequential fashion.

## 5 Discussion

Our results confirm that the fusion between the EMT and visual information using the proposed probabilistic model in a sequential fashion does not accumulate drift. However, there exists an error within the acceptable range of camera poses in which estimates can lay. We believe that the major cause of this error is the use of previous estimates as fixed camera poses, which encourages continuity on subsequent estimations. The effect of this error can be clearly appreciated in Fig. 11(a). In contrast to the PairVis, in which Fig. 8 has drifted after ∼20 frames, our approach is able to create a consistent mosaic after 3770 frames. However, a fixed point in one of the circular loops does not necessarily match to the exact same point when the scene is revisited [Fig. 11(a)]. The range of error experienced corresponds to the spread, where the EMT system allows the estimates to be as long as there is pairwise consistency. To further highlight this fact, we can see that when the estimation exceeds the range of error allowed by the model, an occasional pull toward the true value can be observed (e.g., see frames 18–19). We did not include a version of the LBA using only visual information in our results since it would still use exclusively pairwise visual measurements, and therefore, it would drift.

When the scene is revisited, images are not necessarily stitched in an exactly consistent location. This is a limitation of our approach, and we do not constrain the revisited positions to match. However, an immediate extension that can tackle this problem would consist of the use of a spatiotemporal window to also consider regions of the space that are being revisited. While the probabilistic formulation would remain valid, the spatial window would optimize for loop closures, yet avoiding the computational cost associated with re-estimating the cameras within the loop, since we would rely on the EMT information to situate the loop roughly in a correct location from the beginning.

Our results show how, despite the inherent noise in the EMT system, its measurements can be used to produce accurate pairwise registrations. However, since independence between corresponding points has been assumed in the model and the number of points can be high, if not dealt with, it translates into underestimated uncertainty in the visual information, thus leading to less reliance on the EMT information. Nonetheless, our setup does not yet fully simulate clinical images; the matching process in our datasets is in general easier than in clinical videos, with an increased number of matches, which accentuates the imbalance between both modalities.

The placenta is not completely planar; the violation of this assumption will inevitably produce misregistration errors in those areas, where the nonplanarities are more prominent. Figure 11(c) demonstrates that these errors are small enough to consider the assumption of planarity valid in an *ex vivo* scenario. However, further research should be conducted on *in vivo* data.

Additionally, we see how the feature-based and outlier removal strategy do not perform optimally in *ex vivo* tissue [Fig. 11(c)], which leads to misregistrations in the mosaic. We believe that further research in registration of pairwise images must be done for *ex vivo* and *in vivo* data. However, this is out of the scope of this paper.

If we closely analyze how the noise in the corresponding points is propagated, we see that there are two error terms playing a role in a point $p^{i,B}$ in image B; the corresponding point $p^{i,A}$ in image A gets propagated through the homography, which makes one error term highly correlated between points, and another one is additive, i.e., $p^{i,B}=H(p^{i,A}+\varepsilon^{i,A})+\varepsilon^{i,B}$. Therefore, the distribution already undergoes a nonlinear function, and there can be other nonlinear factors, such as distortion errors that can complicate even more the resulting distribution. For this reason, we opted to approximate such distribution as a Gaussian, which works well in practice.

We have also demonstrated how our approach can perform adequately even when visual information is missing. The reason for this is that we have strong information about missing camera poses: for each empty frame, we have an EMT measurement that tells us the approximate location of the camera, and a temporal model that also provides prior knowledge of its location. Furthermore, the fact that some images are missing does not impede the estimation of the plane as long as the visual information has enough baseline between images.

Our approach estimates the distance d from the reference camera to the surface, which is directly related to the distance between the camera and the surface. This distance can be a powerful cue for the surgeon since photocoagulation must be done at a certain fixed distance from the placenta. However, neither the EMT nor visual information in isolation can estimate such distance. Actually, if no EMT information is provided, the distance becomes a free parameter due to the inherent scale ambiguity in monocular cameras. For this reason, its estimation is not straightforward and further assessment of the accuracy needs to be conducted. Additionally, the hand-eye calibration is in general problematic; a set of images of a known object, e.g., a checkerboard, must be taken with the fetoscope and the attached EMT sensor in controlled conditions. One may also need to apply some heuristics to achieve an acceptable accuracy.

## 6 Conclusions

We have presented a probabilistic model for robust drift-free sequential mosaicking that fuses imagery and data from an EMT system in the case where a planar or quasiplanar object is imaged in a hand-held motion. We have shown that our method does not accumulate error; a problem that affects all monocular pairwise mosaicking systems, which use exclusively visual information. Therefore, we have been able to create long mosaics obtaining an accuracy comparable to the state-of-the-art bundle adjustment. In spite of the inherent noise in EMT systems, we have demonstrated that our approach can still generate accurate mosaics while leveraging its guidance. Furthermore, we have shown its feasibility even when there is a loss of visual information.

In terms of future work, we are considering the following research lines: (i) clinical fetoscopic datasets present a challenge in terms of obtaining a set of valid matches. In this work, we have not addressed this problem; however, this is clearly a limitation toward fetoscopic mosaicking that we plan to address. (ii) In order to eliminate the error within the EMT bounds, we could consider the use of weighted LBA. This would soften the effect of fixed cameras and reconsider already estimated cameras according to their uncertainty in the current estimation. (iii) To further avoid normalization in the corresponding points, modeling the fact that the errors in the points are correlated is an interesting idea, which could avoid the normalization process. (iv) The inclusion of the visual and hand-eye matrix in the model is an attractive research line, which, if successful, would remove a tedious step in the operating room. (v) Last, this study serves as a proof-of-concept to a future real-time version of the approach. We believe this is possible given that algorithms with similar computational load have been successfully implemented in real time.

Given the low quality of fetoscopic images, we believe that the inclusion of the EMT system in the mosaicking process is fundamental to achieve a robust and accurate mosaic, independently of the number of frames.

## Appendix A

### A1 Covariance Matrices

$$\Sigma_{\mathrm{EMT}}=\mathrm{diag}([0.017,0.017,0.017,1,1,1]),\;\Sigma_{p}=\mathrm{diag}([0.0044,0.0044,0.0044,15.2178,15.2178,15.2178]),$$ (30)

where diag(·) denotes a diagonal matrix.

### A2 Conversion from Cartesian Coordinates to Azimuth, Elevation, and Distance

The conversion from Cartesian coordinates to azimuth, elevation, and distance is as follows:

$$\tan(az)=\frac{y}{z},\;\tan(\mathrm{elev})=\frac{x}{\sqrt{z^{2}+y^{2}}},\;d=\sqrt{z^{2}+y^{2}+z^{2}}.$$ (31)
